# Intravascular Ultrasound (IVUS)-Assisted Fenestrated Endovascular Aortic Repair (FEVAR) for Iuxtarenal Aortic Aneurysm with Pelvic Ectopic Left Kidney and Aberrant Left Renal Arteries: A Case Report

**DOI:** 10.3390/reports9040326

**Published:** 2026-09-27

**Authors:** Priscilla Nardi, Giulia Guadalupi, Valerio Rinaldi, Edoardo De Meis, Valeria Iacoucci, Greta D’Onofrio, Eleonora Zedda, Rocco Pasqua, Fabio Miraldi, Gaetano Tanzilli, Nicola Viceconte, Francesco Pelliccia, Giampaolo Prezioso, Vito D’Andrea, Giulio Illuminati

**Affiliations:** 1 Department of Surgery, Sapienza University of Rome, Viale Regina Elena, 324, 00161 Rome, Italy; 2 Department of Interventional Cardiology, Sapienza University of Rome, Viale Regina Elena, 324, 00161 Rome, Italy; 3 Department of Cardiac Surgery, Sapienza University of Rome, Viale Regina Elena, 324, 00161 Rome, Italy

**Keywords:** IVUS, FEVAR, endovascular, case report, aberrant vascular anatomy, complex aneurysm, embolization

## Abstract

**Background and Clinical Significance**: Complex abdominal aortic aneurysms (AAAc) involving visceral and renal vessels can be treated via open surgery or less invasive fenestrated endovascular aneurysm repair (FEVAR). Intravascular ultrasound (IVUS) minimizes contrast and radiation while improving placement accuracy, though its specific role in FEVAR remains understudied. This CARE-compliant article reports a FEVAR case guided by IVUS. **Case Presentation**: A 75-year-old male with multiple comorbidities presented with an enlarging AAAc extending to the renal artery ostia, lacking an adequate neck. Anatomical variants included a left renal artery with a caudal origin (pelvic left kidney), a left renal polar artery originating from the right common iliac artery, and a patent inferior mesenteric artery (<3 mm). Following multidisciplinary evaluation, the patient successfully underwent simultaneous FEVAR and coronary angiography. Guided by IVUS, the fenestrated endograft was deployed with preserved visceral patency. The patient recovered uneventfully and was discharged on postoperative day two. A 30-day CT angiogram detected a stable inferior mesenteric artery endoleak, managed conservatively through a 6-month follow-up. **Conclusions**: IVUS-guided FEVAR may offer a promising alternative to traditional angiography, reducing radiation and contrast exposure. Further large-scale studies are needed to confirm these findings and establish clinical and economic benefits.

## 1. Introduction and Clinical Significance

Complex abdominal aortic aneurysms (AAAc) involve the aortic segment supplying the kidneys and viscera, categorized into short-neck infrarenal, juxtarenal, pararenal, paravisceral, and type IV thoracoabdominal aneurysms [1,2,3]. Co-occurrence of complex abdominal aortic aneurysms (AAAc) and pelvic ectopic kidneys (<0.1 of cases) presents significant anatomical challenges due to aberrant renal blood supply originating from the distal aorta or iliac vessels. While open surgical repair (OSR) provides excellent long-term durability, it carries substantial perioperative morbidity and mortality in high-risk patients due to extensive surgical exposure, supraceliac clamping, and risk of permanent renal ischemia. Conversely, fenestrated endovascular aneurysm repair (FEVAR) has emerged as a safe, minimally invasive alternative that achieves complete sac exclusion while preserving visceral and ectopic renal perfusion via customized fenestrations and bridging stents. Although FEVAR significantly reduces early mortality, morbidity, and hospital stays compared to OSR, it requires meticulous multiplanar CT planning, high technical expertise, and mandatory long-term surveillance to monitor for endoleaks and target-vessel instability. Ultimately, treatment choice must balance patient surgical risk, anatomical suitability, and institutional expertise [1,4,5,6,7,8,9,10]. FEVAR demonstrates proven short- and mid-term safety, high target vessel perfusion (90–100%), low 30-day mortality (<4%), minimal endoleaks and migration (<3%), and kidney dysfunction as a primary complication, making it valuable for high-risk patients [11]. Intravascular ultrasound (IVUS) uses a catheter-based miniaturized probe to generate detailed vessel images, minimizing contrast and radiation, ensuring safety, and enabling precise intraoperative measurements of aortic diameters and neck lengths [3,12]. Although IVUS is well-established in EVAR, TEVAR, and dissections, its specific role and benefits in FEVAR remain less extensively studied [12,13,14].

Clinical Significance: This study highlights that IVUS-guided FEVAR could be a safe and effective alternative for complex AAAc repair, reducing contrast media and radiation exposure. This approach is particularly advantageous for high-risk patients with renal impairment, offering precise endograft placement and favorable mid-term outcomes. While IVUS utility in standard EVAR is established, its application during FEVAR in the setting of severe anatomical variants—specifically a pelvic ectopic kidney with aberrant renal circulation—combined with simultaneous coronary evaluation. The main novelty of this case rests on the synergistic management of these three complex clinical challenges.

## 2. Case Presentation

The article was written in accordance with the CARE guidelines for writing case reports [15]. Informed consent for the surgical procedure was obtained from the patient, which also included consent to treat his case in full respect of privacy for scientific purposes.

A 75-year-old male patient presented to the vascular surgery team at Policlinico Umberto I (Rome, Italy) for an increase in size (>1 cm in 1 year) of a previously known abdominal aortic aneurysm, present in his medical history for approximately 1 year. Details regarding the patient’s history and physical examination are provided in Table 1 and Table 2.

Pre-hospitalization tests were largely normal, except for mild anemia (Hb: 10.4 g/dL). Renal function was preserved (GFR: 90 mL/min). A chest X-ray showed subpleural parenchymal opacities and calcifications consistent with prior sequelae. Doppler ultrasound revealed non-significant bilateral carotid and lower limb stenosis due to mild atheromasia.

The abdominal aortic aneurysm had a maximum transverse diameter of 5.12 cm and involved the ostia of the renal arteries, lacking sufficient neck for the digestive arteries. Additionally, the left renal artery had a low origin due to the presence of a pelvic left kidney. A left renal polar artery originating from the right common iliac artery, with a diameter > 4 mm, was also present, and consequently, an embolization intervention was required. The inferior mesenteric artery was patent with a diameter < 3 mm. The iliac-femoral axis exhibited mild calcific parietal atheromasias without bilateral stenotic lesions. Figure 1 shows the 3D reconstruction of the complex aneurysm on CT angiography.

The patient’s case was brought to the attention of a multidisciplinary team composed of a surgeon, cardiologist, interventional cardiologist, arrhythmologist, and anesthesiologist. In light of the patient’s significant comorbidities, endovascular repair was deemed the optimal strategy after discussing all available treatment options, including open surgical repair, with the patient. It was decided to submit the patient to simultaneous FEVAR and coronary angiography.

Following an arrhythmology consultation, the pacemaker was successfully reprogrammed (and adjusted again on the second postoperative day), and the Cardicor dosage was increased to 5 mg daily. Pradaxa was discontinued 3 days preoperatively and bridged with subcutaneous Enoxaparin (100 IU/kg/day) due to the patient’s high risk of thromboembolism. Enoxaparin was paused the morning of surgery, resumed for two days postoperatively, and then successfully transitioned back to Pradaxa. To prevent lactic acidosis, metformin was stopped 48 h prior to the procedure. A rapid-acting insulin sliding scale was established upon admission, though administration was never required.

The procedure was performed under general anesthesia (ASA 3) by a multidisciplinary team (vascular surgeons and interventional cardiologists). Preoperative protocols included an 8 h fast, intravenous Cefazolin (2 g) prophylaxis, right radial artery line placement for invasive blood pressure monitoring, and a urinary catheter. Access sites (femoral bifurcations and left brachial artery) were mapped via ultrasound before positioning the patient.

Percutaneous puncture of the left femoral artery and left brachial artery and insertion of guidewires and introducers were performed, followed by systemic heparinization. The heparinization scheme was 5000 IU after introducer insertion + 2500 IU after 150 min from the intervention’s start + 2500 IU after 270 min, of which 2500 IU were neutralized at the end of the procedure. The first phase of the operation was characterized by the execution of coronary angiography, which revealed non-obstructive coronary atheromatosis (stenoses < 40%), requiring no immediate coronary intervention, thereby allowing us to proceed directly with the endovascular repair. Subsequently, cannulation of the left accessory renal artery originating from the right common iliac artery and embolization of the same using an 8 mm wax plug (Boston Scientific, Marlborough, MA, USA) was carried out. The surgical procedure continued with identification of the ostia of the celiac trunk, superior mesenteric artery and renal arteries by 0.035” IVUS probe (Volcano Vision R PV 8.2 French; Volcano Japan Inc., Tokyo, Japan) marking them on the C-arm screen (Figure 2).

A Fenestrated Treo 4 bottom endograft (Terumo Aortic, Elk Grove Village, IL, USA) was implanted via the right femoral access. The main prosthetic body was positioned, aligning the fenestration markers with the markers on the screen as visualized by IVUS. The prosthesis was partially deployed, and the superior mesenteric artery (via the left brachial access) and the left renal artery (via the left femoral access) were cannulated. Only after confirming correct placement was the prosthesis fully deployed and Begraft stents (Bentley InnoMed GmbH, Langen, Deutschland) were placed in the celiac trunk (7 × 27 mm), superior mesenteric artery (8 × 37 mm), and both renal arteries (7 × 57 mm right, 7 × 37 mm left). Iliac extensions Bolton Treo were also implanted. Expansion of the prosthetic legs with balloons was performed. Table 3 shows the step-by-step IVUS-guided FEVAR workflow. 

Since the inferior mesenteric artery had a diameter of <3 mm, it was not routinely embolized [1]. The procedure was successful, with satisfactory arteriographic control (patency of celiac trunk, superior mesenteric artery, right and left renal arteries and hypogastric arteries), correct embolization of the left accessory renal artery and no visible endoleak. The procedure was successful, with satisfactory arteriographic control (patency of celiac trunk, superior mesenteric artery, right and left renal arteries and hypogastric arteries), correct embolization of the left accessory renal artery and no visible endoleak. Hemostasis of femoral arteries was achieved with 2 Pro-glide (Abbott Laboratories, Abbott Park, IL, USA) on the right and two on the left, and by compression of the left brachial artery. The one-centimeter femoral skin incisions were closed with 2-0 Nylon sutures. The patient’s lower limbs and left upper limb were warm with palpable distal pulses at the end of the procedure, and a compressive dressing was placed on the femoral and brachial access sites.

The surgery lasted 345 min, the fluoroscopy time was 55 min, and the amount of contrast medium used was 40 mL. The patient was awakened and extubated in the operating room and transferred to the intensive care unit for intensive monitoring for at least the first 12 postoperative hours, during which no complications occurred. For these reasons, on the first postoperative day, he was transferred to the surgical ward, the urinary catheter was removed, and feeding was resumed. Following the absence of complications at the vascular access sites, the compressive dressings were removed 24 h after the end of surgery. The patient had no fever or increase in white blood cells or inflammatory markers and no acute renal failure (postoperatively, renal function remained baseline-stable, serum creatinine at discharge was 0.92 mg/dL, at 30-day follow-up was 0.95 mg/dL, and at 6-month follow-up remained at 0.91 mg/dL). Asymptomatic anemia (serum hemoglobin: 8.7 g/dL) was observed on the first postoperative day, which did not require blood transfusions or supplements through intravenous iron administration. The patient was discharged in good general condition and independently ambulating on the second postoperative day with instructions to resume his usual home therapy.

Thirty days after the surgical procedure, the patient underwent a follow-up abdominal CT angiogram which showed correct positioning of the fenestrated endograft, patency of the visceral vessels, correct embolization of the accessory renal artery, and absence of complications from the femoral access sites; however, it should be noted that an endoleak from reperfusion of the aneurysmal sac, currently measuring 50 × 51 mm, originating from the inferior mesenteric artery (IMA) (Figure 3). 

Since the aneurysmal sac has not increased in size, it was decided to monitor the endoleak over time. Abdominal aortic echo-color Doppler ultrasound was performed at 1 month and then at 6 months after the CT scan, which showed stability of the aneurysmal sac. At the 12-month follow-up, abdominal duplex ultrasound demonstrated complete stability of the aneurysmal sac (50 × 51 mm) with low-flow Type II endoleak persistence, patency of all visceral and renal target branches, preserved renal function, and total absence of graft-related complications or secondary reinterventions. It was therefore recommended that the patient continue to undergo regular checkups every 6 months.

## 3. Discussion

The average operative time for a FEVAR procedure is highly variable—the literature reports average durations ranging from 100 to 450 min, the amount of contrast media used varies from 30 to 120 mL, and the fluoroscopy time ranges from 20 to 90 min. The rate of type II endoleak occurrence at 1 month after the procedure is not well defined in the literature (it can vary from 10 to 30%). However, although it is not possible to provide a precise number, it is important to be aware of the risk of its occurrence and to carry out careful follow-up [16,17].

The duration of the procedure, the volume of contrast agent administered, and the fluoroscopy time employed in this case are all within the ranges reported in existing medical literature for similar endovascular interventions. However, it is crucial to acknowledge that the reported operative times, contrast usage, and fluoroscopy exposure reflect the completion of two separate and distinct procedures during the same surgical session.

Diagnostic and potentially therapeutic coronary angiography may be indicated in patients with AAA requiring surgical treatment, as 40% to 60% of patients with AAA also have coronary artery disease (CAD). Failure to diagnose significant coronary lesions may lead to complications in these patients that can benefit from the safety and efficacy of simultaneous percutaneous coronary intervention (PCI) and EVAR. This combined procedure addresses both conditions at once, eliminating the risks associated with separate surgeries and delays in treatment and reducing the risk of myocardial infarction in the perioperative period [18]. In our decision-making strategy, if significant CAD requiring PCI had been identified, the clinical plan was to perform PCI first. Had PCI been completed successfully without complications or hemodynamic instability, FEVAR would have proceeded within the same session. Conversely, in the event of intraoperative PCI complications or instability, FEVAR would have been staged for a later date.

IVUS utilization allows contrast volume optimization. Performing simultaneous coronary angiography and FEVAR in a single operative setting delivers a high cumulative nephrotoxic load.

Utilizing IVUS as an adjunct imaging tool allowed us to achieve complete visceral vessel identification while restricting total contrast media consumption. Preventing contrast-induced nephropathy through contrast-sparing techniques is beneficial regardless of baseline renal status, particularly during complex multi-stage or combined endovascular interventions.

Carbon dioxide (CO_2_) angiography was not utilized because of limitations regarding image resolution during delicate visceral branch cannulation and risk of arterial gas embolism or transient severe abdominal discomfort [19,20].

One of the main advantages of IVUS is its ability to provide precise measurements of aortic and iliac diameters. The IVUS probe is centimeter-marked, which means that during its retraction, the distances traveled inside the vessel can be accurately measured. This feature allows for measuring the diameter of the aorta and iliac arteries at different points, measuring the length of specific segments of the aorta and iliac arteries, precisely visualizing the aortic bifurcation, the iliac bifurcation and the visceral vessel ostia, fundamental landmarks during endovascular procedures. Measurements obtained with IVUS during an endovascular procedure can be compared with those previously performed using other imaging techniques, such as computed tomography (CT) angiography. This allows us to verify the accuracy of preoperative measurements and, if necessary, to modify the length of the prosthetic legs or choose stents of different sizes to ensure an optimal fit [20].

IVUS is also a valuable tool for assessing the correct placement of stents. It is useful to verify complete stent expansion, ensuring that the stent has opened correctly inside the vessel, without areas of compression or malposition, checking that the stent adheres uniformly to the vessel walls, without gaps that could promote the formation of blood clots. It can promptly identify any complications such as dissections and assess stent stability, especially in the presence of complex anatomies or calcifications. Accurate stent placement and good stability are essential to ensure vessel patency [21].

A learning curve is necessary to use this technique correctly and get the biggest benefit; the interpretation of IVUS images requires some experience and expertise on the part of the operator, and this can add time to the procedure, especially in the early stages of learning. However, with experience, this additional time can be reduced. In our experience, the absence of a dedicated hybrid suite was one of the clinical drivers for implementing IVUS during FEVAR. Nevertheless, this choice was not only a reaction to structural constraints. IVUS offers several cost advantages compared to a hybrid room setup for endovascular procedures. While IVUS requires a single-use catheter and ultrasound unit (lower initial cost than an angiograph), hybrid rooms necessitate a substantial investment in angiographic equipment, integrated tables, high-definition screens, and other specialized tools. IVUS equipment and personnel costs are also less expensive than for the complex systems in a hybrid room. Finally, IVUS can reduce indirect costs associated with contrast media (less expensive and nephrotoxic) and potential complications [21].

## 4. Conclusions

Utilizing IVUS during FEVAR may potentially assist in reducing exposure to radiation and contrast media, which could offer nephroprotective benefits for patients with chronic kidney disease; however, these clinical benefits require formal confirmation through larger comparative studies. While this approach may offer potential resource and cost advantages by facilitating complex endovascular repairs outside of dedicated hybrid operating suites, larger multi-center studies are required to evaluate procedural costs, healthcare resource utilization, and long-term feasibility across different clinical settings.

## Figures and Tables

**Figure 1:**
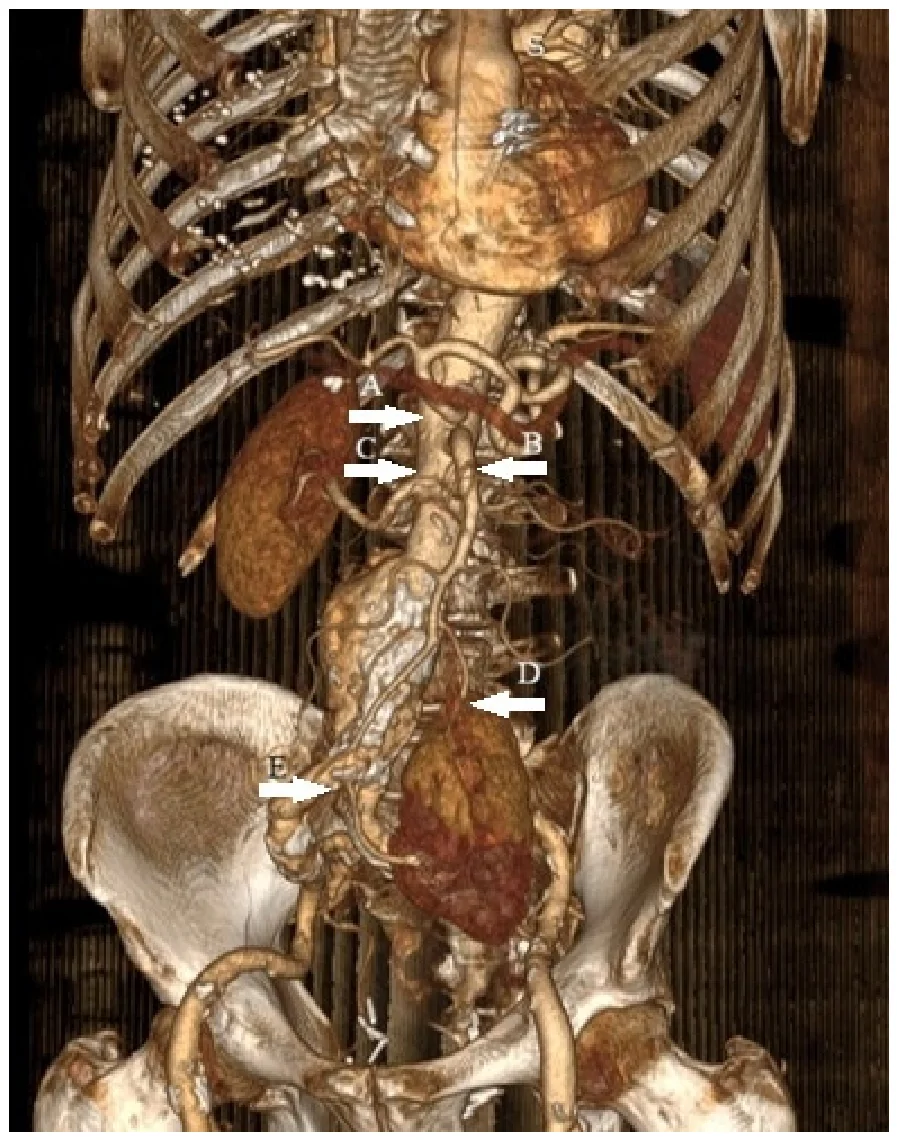
3D reconstruction of the complex aneurysm on CT angiography. (**A**) Celiac trunk; (**B**) superior mesenteric artery; (**C**) right renal artery; (**D**) left renal artery; (**E**) left polar renal artery originating from right common iliac artery.

**Figure 2:**
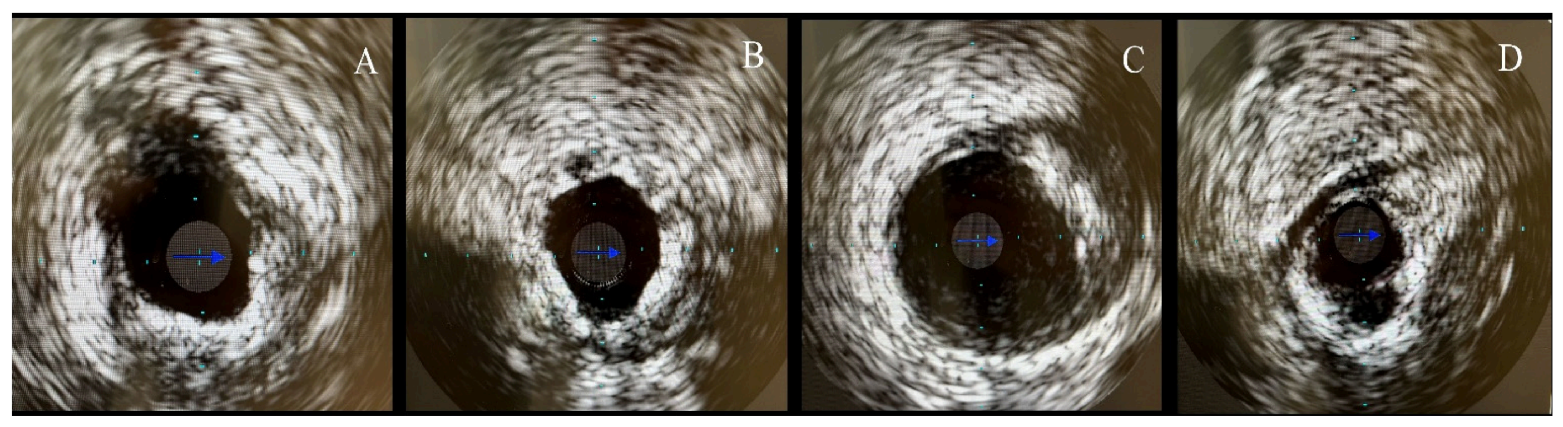
Intraoperative IVUS representation of the identification of the celiac artery ostium (**A**), inferior mesenteric artery (**B**), abdominal aortic aneurysm (**C**) and external and internal iliac bifurcation (**D**).

**Figure 3:**
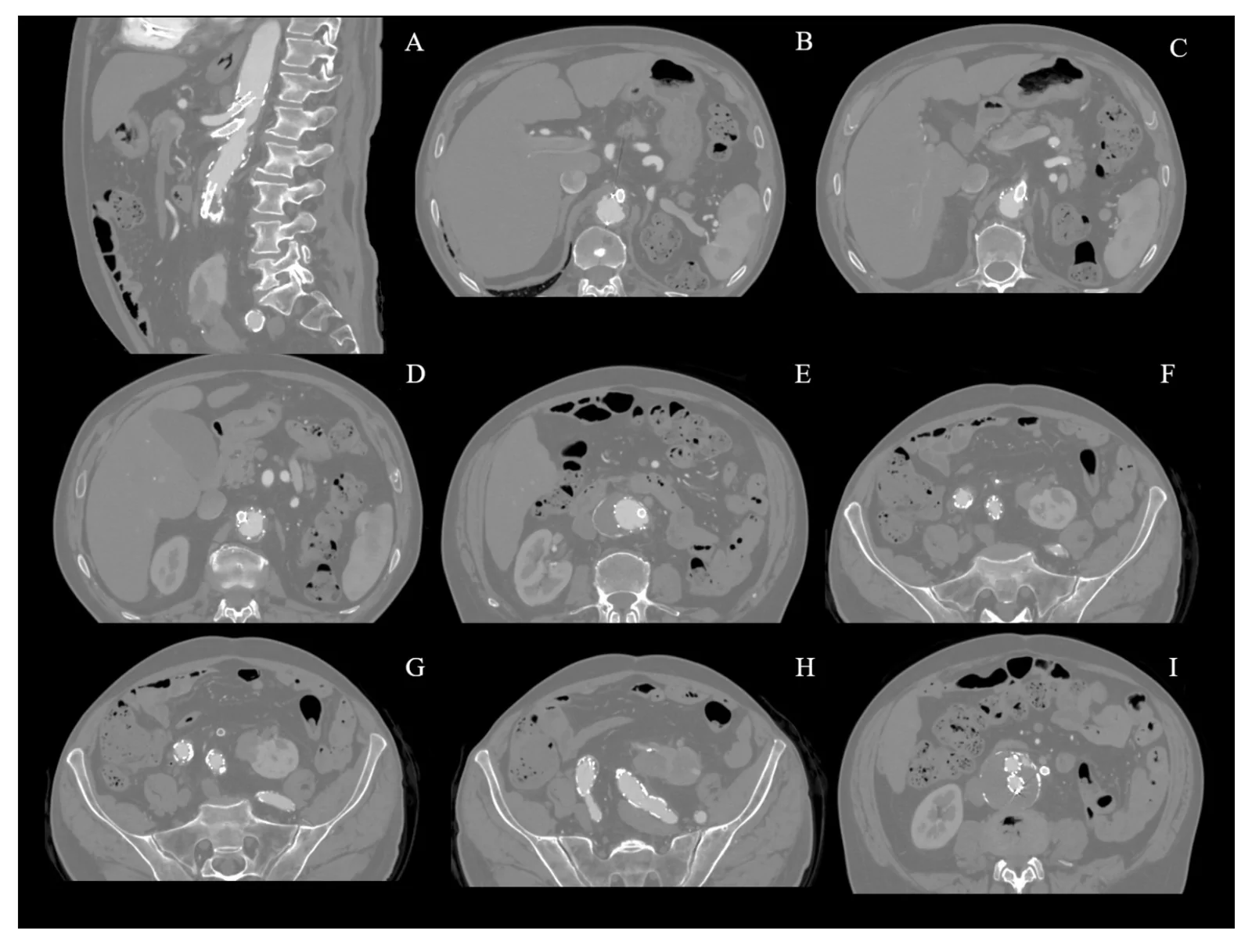
Postoperative CT angiography representation showing, in the sagittal section (**A**) and axial section, the patency of the celiac arteries (**B**) and superior mesenteric artery (**C**), the right renal artery (**D**), the left renal artery with caudal origin (**E**), the correct embolization of the left renal polar artery (**F**) and the plug within it (**G**), the bilateral external and internal iliac bifurcation (**H**), and the type II endoleak originating from the inferior mesenteric artery (**I**).

**Table 1:** Patient history.

**Family history**	Significant for cerebral aneurysm. No other significant findings.
**Medical history**	
**Cardiovascular**	Hypertension Dyslipidemia Atrial fibrillation Atrioventricular (AV) block requiring dual-chamber pacemaker implantation Hospitalization for acute heart failure Non-significant diffuse calcific atheromasias of the carotid and femoral arteries
**Neurological/Cerebrovascular**	Stroke Transient Ischemic Attack (TIA)
**Metabolic/Endocrine**	Type 2 diabetes mellitus (complicated by diabetic polyneuropathy) Hepatic steatosis
**Respiratory System**	Pneumoconiosis Hospitalization for pneumonia
**Musculoskeletal**	Cervical and lumbar discopathy
**Peripheral Vascular**	Venous insufficiency of the lower limbs, complicated by left great saphenous vein (GSV) incompetence
**Current medications**	Metformin, Ramipril, Furosemide, Pregabalin, Dabigatran, Simvastatin, Allopurinol, Incretin
**Allergies**	-
**Lifesyle**	Smoking: Significant history (40 cigarettes/day for 50 years; quit 13 years ago) Alcohol: Denies any history of consumption. Substance Abuse: Denies any history.
**Surgical history**	Left orchiectomy Radical prostatectomy

**Table 2:** Physical examination.

	Examination Findings
**Musculoskeletal**	Hypotonic and hypotrophic muscle mass. Mobility is impaired by pain due to diabetic neuropathy and multiple disc protrusions.
**Abdominal examination**	Globose abdomen due to adiposity. A pulsatile mass (approximately the size of an orange) was palpable in the mesogastric region.
**Vascular examination**	Carotid, subclavian, brachial, radial, femoral, popliteal, posterior tibial, and dorsalis pedis pulses are palpable and of normal volume bilaterally. No pathological bruits auscultated.

**Table 3:** Step-by-Step IVUS-Guided FEVAR Workflow.

**Phase 1: Pre-deployment Mapping**: manual 0.035” IVUS catheter pullback from thoracic aorta to iliac bifurcations to identify visceral artery ostia.
**Phase 2: Fluoroscopic co-registration**: markers of visceral artery ostia transferred directly onto the C-arm fluoroscopy screen without digital fusion software.
**Phase 3: Graft alignment & cannulation**: alignment of fenestrated endograft main body markers with screen-mapped ostial locations under fluoroscopic and IVUS control, followed by target branch cannulation.
**Phase 4: Completion assessment**: post-deployment IVUS pullback across all target branches and main body to confirm stent expansion, wall apposition, and rule out mechanical complications or dissections.

## Data Availability

The data presented in this study are available on request from the corresponding author. The data are not publicly available due to privacy and ethical restrictions.

## Abbreviations

The following abbreviations are used in this manuscript:

IVUS	IntraVascular UltraSound
FEVAR	Fenestrated Endovascular Aortic Repair
EVAR	Endovascular Aortic Repair
TEVAR	Thoracic Endovascular Aortic Repair
PCI	Percutaneous Coronary Intervention
AAA/AAAc	Abdominal Aortic Aneurysm/Complex Abdominal Aortic Aneurysm
IMA	Inferior Mesenteric Artery
CAD	Coronary Artery Disease
TIA	Transient Ischemic Attack
GSV	Great Saphenous Vein
AV Block	Atrioventricular Block
CT	Computed Tomography
GFR	Glomerular Filtration Rate
IU	International Units
CARE	CAse REport guidelines
ASA	American Society of Anesthesiologists
Fr	French
Hb	Hemoglobin
